# Low Citrate Synthase Activity Is Associated with Glucose Intolerance and Lipotoxicity

**DOI:** 10.1155/2019/8594825

**Published:** 2019-03-03

**Authors:** Yosra Alhindi, Lobke M. Vaanholt, Mustafah Al-Tarrah, Stuart R. Gray, John R. Speakman, Catherine Hambly, Bader S. Alanazi, Brendan M. Gabriel, Arimantas Lionikas, Aivaras Ratkevicius

**Affiliations:** ^1^Clinical Pharmacy Department, Pharmacy Collage, Umm Al-Qura University, Makkah, Saudi Arabia; ^2^Institute of Biological and Environmental Sciences, University of Aberdeen, Aberdeen, UK; ^3^School of Medicine, Medical Sciences and Nutrition, University of Aberdeen, Aberdeen, UK; ^4^Institute of Cardiovascular and Medical Sciences, University of Glasgow, Glasgow, UK; ^5^Karolinska Institute, Department of Physiology and Pharmacology, Integrative Physiology, Stockholm, Sweden; ^6^Department of Applied Biology and Rehabilitation, Lithuanian Sports University, Sporto 6, Kaunas LT 44221, Lithuania

## Abstract

Citrate synthase (CS) is a key mitochondrial enzyme. The aim of this study was to test the hypothesis that low CS activity impairs the metabolic health of mice fed a high fat diet (HFD) and promotes palmitate-induced lipotoxicity in muscle cells. C57BL/6J (B6) mice and congenic B6.A-(rs3676616-D10Utsw1)/KjnB6 (B6.A), a strain which carries the A/J allele of CS on the B6 strain background, were fed HFD (45% kcal from fat) for 12 weeks. C2C12 mouse muscle cells were used to investigate effects of CS knockdown on cell viability and signalling after incubation in 0.8 mM palmitate. CS activity, but not that of *β*-hydroxyacyl-coenzyme-A dehydrogenase was lower in the gastrocnemius muscle and heart of B6.A mice compared to B6 mice (*P* < 0.001). During HFD feeding, glucose tolerance of mice decreased progressively and to a greater extent in B6.A females compared to B6 females, with males showing a similar trend. Body weight and fat gain did not differ between B6.A and B6 mice. After an 18 h incubation in 0.8 mM palmitate C2C12 muscle cells with ∼50% shRNA mediated reduction in CS activity showed lower (*P* < 0.001) viability and increased (*P* < 0.001) levels of cleaved caspase-3 compared to the scramble shRNA treated C2C12 cells. A/J strain variant of CS is associated with low enzyme activity and impaired metabolic health. This could be due to impaired lipid metabolism in muscle cells.

## 1. Introduction

Mitochondria play a key role in functioning of skeletal muscles and metabolic health [1, 2]. Insulin resistance often coincides with reduced mitochondrial oxidative capacity in skeletal muscle [3]. Mitochondrial citrate synthase (CS) has often been used as a biomarker of mitochondrial content and function in mammals [4, 5]. However, we have recently reported that CS activity in skeletal muscles of A/J mice is reduced by half compared to five other strains of mice [6, 7]. As neither abundance of the CS protein nor other mitochondrial markers could explain this reduction, we attributed this phenomenon to the missense mutation in exon 3 of *Cs*, i.e., H55N substitution (A for C, rs29358506) in the A/J mice. Thus, mice carrying the A/J allele of *Cs* could be a prudent model for studying the effects of reduced CS activity on such conditions as obesity and diabetes. This is important in view of the evidence suggesting that innate impairment in CS functioning might be responsible for low rates of fatty acid oxidation and insulin resistance in skeletal muscles of diabetic patients [8, 9]. If CS plays a causative role, muscle cells with low CS activity will show accelerated apoptosis after prolonged incubation in the media enriched with palmitate which induces lipotoxicity in muscle cells [10]. Similarly, mice carrying the A/J allele of *Cs* will be affected by a more severe impairment in glucose tolerance than the mice carrying the wild type or C57BL/6J (B6) strain allele of *Cs* when fed high fat diet (HFD) which is known to induce insulin resistance [11]. Interestingly, A/J mice are more resistant to fat and weight gain compared to the obesity prone B6 strain [12, 13]. This difference in obesity resistance between the strains might also be associated with CS activity since the chromosome substitution strain, which carries chromosome 10 of the A/J strain on B6 strain background, is also obesity resistant [14]. Furthermore, a congenic strain carrying the A/J allele in the telomeric region of chromosome 10, where the *Cs* gene resides, showed higher resistance to obesity than B6 mice [15, 16]. All these findings prompted us to investigate the role of the H55N polymorphism in physiological adaptations to HFD.

The aim of the study was to test the hypothesis that the H55N polymorphism affects CS activity and metabolic health in mice fed HFD. We compared metabolic and physiological responses to a HFD in B6 mice and congenic B6.A-(rs3676616-D10Utsw1)/KjnB6 (B6.A) mice which carry the A/J allele of the *Cs* gene on the B6 background [17]. We have also tested the hypothesis about the link between low CS activity and impairment in lipid metabolism and lipotoxicity in muscle cells. In these experiments, we examined effects of reduced *Cs* expression on muscle cell viability; ATP levels and production of reactive oxygen species (ROS) were measured before and after incubation in the media supplemented with high concentrations of palmitate which induces apoptosis [10].

## 2. Results

### 2.1. Chromosome 10 Genotype Is Associated with CS but Not HAD Activity

**Figure 1:**
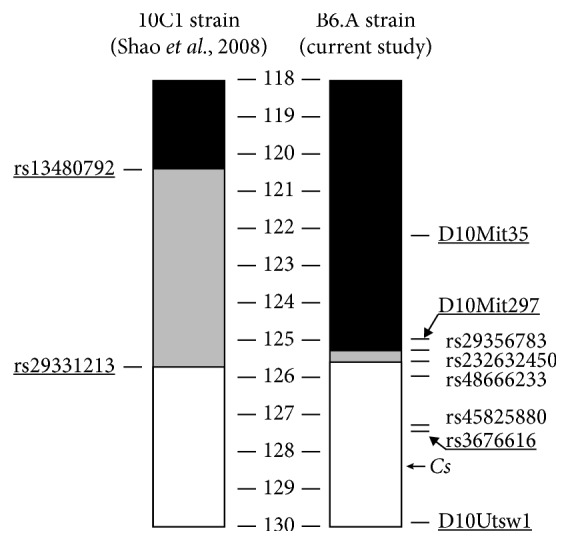
Genotyping chromosome 10 in congenic B6.A-(rs3676616-D10Utsw1)/KjnB6 (B6.A) strain. Black, white, and gray colours mark alleles of C57BL/6J (B6), A/J genotype, and unknown genotype, respectively. The chromosomal markers are also indicated. The data are also presented for congenic (10C1) strain in [16].

The proximal region of the chromosome 10 was genotyped in order to refine the recombination site in the congenic B6.A strain (Figure 1). This analysis confirmed that congenic B6.A mice carried A/J allele of *Cs*. We compared enzyme activity for CS and HAD in the gastrocnemius muscle and heart of B6 and B6.A mice at the end of HFD study (Table 1). Activity of these enzymes, especially that of HAD, was higher (*P* < 0.001) in the heart compared to the gastrocnemius muscle. For both tissues, CS activity was also higher (*P* < 0.001) in B6 mice than in B6.A mice, but there were no strain effects on HAD. ANOVA showed that CS and HAD activity in the gastrocnemius muscle depended on sex (*P* < 0.05) as females tended to have lower activity of these enzymes, but there were no such sex effects on the heart.

### 2.2. HFD Feeding Leads to a Greater Gain in Fat Mass of Male Mice than Female Mice

**Figure 2:**
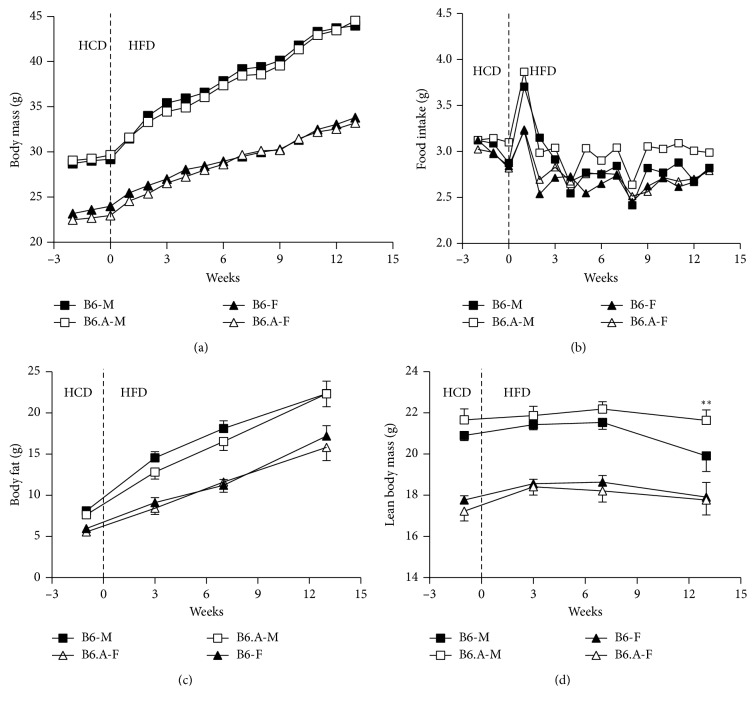
Body mass and composition of mice. Body mass (a), food intake (b), body fat, (c) and lean body mass (d) in C57BL/6J (B6) and congenic B6.A-(rs3676616-D10Utsw1)/KjnB6 (B6.A) mice (M, males; F, females) during the baseline period of high carbohydrate diet (HCD) followed by high fat diet (HFD) feeding. Values (*n*=16 each) are means in A and B as well as means ± SEM in (c) and (d) ^*∗∗*^*P* < 0.01, B6 vs B6.A strains.

Data on body mass and food intake in HFD study are presented in Figure 2. Male mice were always heavier (*P* < 0.001) than female mice, but there were only minor strain effects on body weight. During the first two weeks of HFD feeding, B6 males gained more weight than B6.A males (0.342 ± 0.069 vs 0.261 ± 0.104 g/day, *P* < 0.05, respectively), but there were no differences between the strains among females (0.164 ± 109 vs 0.173 ± 117 g/day, respectively) which gained less (*P* < 0.001) weight than males. Afterwards, the rate of weight gain slowed down (*P* < 0.001), but males continued to gain more weight than females (*P* < 0.01) though there were no differences between the strains. Similarly, food intake showed neither effects of strain nor sex. Food intake increased (*P* < 0.001) during the first week of HFD feeding and then returned to the original level. Fat mass of mice increased continuously (*P* < 0.001) and was smaller in females compared to males (*P* < 0.01), but did not differ between the strains. LBM showed only small changes compared fat mass. B6.A females experienced a small increase (*P* < 0.05) in LBM after the two weeks of HFD feeding and B6 males showed a decrease (*P* < 0.01) in LBM during the last six weeks of the feeding. Males had bigger (*P* < 0.05) LBM than females, but there were no effects of the strain on LBM.

### 2.3. Energy Balance and Resting Metabolic Rate Are Not Dependent on Sex, Strain, or Diet of Mice

**Figure 3:**
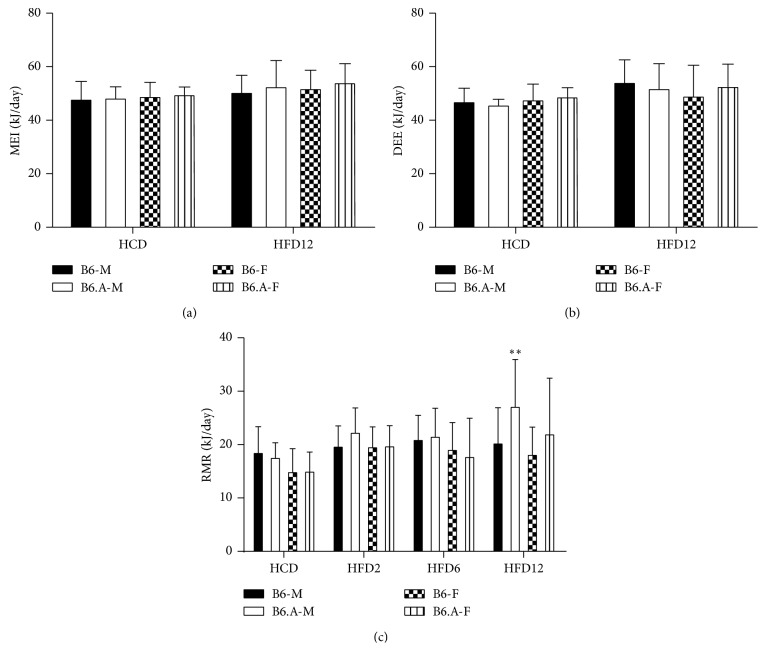
Energy balance and resting metabolic rate of mice. Metabolisable energy intake (MEI) (*n*=8 each) (a), daily energy expenditure (DEE) (*n*=8 each) (b), and resting metabolic rate (RMR) (*n*=16 each) (c) in C57BL/6J (B6) and congenic B6.A-(rs3676616-D10Utsw1)/KjnB6 (B6.A) mice (M, males; F, females) fed high carbohydrate diet (HCD) and high fat diet (HFD). Values are means ± S.D. ^*∗∗*^*P* < 0.05, B6 vs B6.A strains.

Data on energy balance in the HFD study are presented in Figure 3. The covariance analysis, using body mass as a covariate, did not show any significant effect of strain, sex, or diet on MEI or DEE. These factors did not affect RMR either except at the 12 weeks of HFD feeding when B6.A males showed higher RMR compared to B6 males (*P* < 0.01).

### 2.4. Physical Activity and Body Temperature

**Figure 4:**
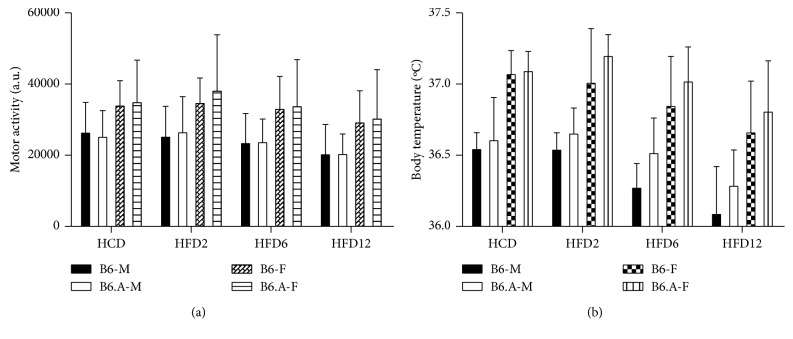
Physical activity and body temperature. Average daily physical activity (a) and body temperature (b) in C57BL/6J (B6) and congenic B6.A-(rs3676616-D10Utsw1)/KjnB6 (B6.A) mice (M, males; F, females). The mean values for physical activity during the light phase are marked by white colour. The data are presented for the baseline period of high carbohydrate diet (HCD) feeding followed by HFD feeding for 2 weeks (HFD2), 6 weeks (HFD6), and 12 weeks (HFD12). Values are means ± SD (*n*=16 each).

The data on physical activity and body temperature of B6 and B6.A mice in HFD study are shown in Figure 4. Physical activity was not affected by strain, but was higher for female mice compared to males (*P* < 0.001). Mice of both sexes became less active (*P* < 0.05) during HFD feeding. The activity during the light phase and dark phase tended to be equally affected. Body temperature was also higher (*P* < 0.001) in females than males and also decreased (*P* < 0.05) during HFD feeding. B6.A males had higher (*P* < 0.01) body temperature during HFD feeding compared to B6 males, but the strain effect was not significant for females.

### 2.5. Tissue Fat

Data on tissue fat at the end of HFD study are presented in Table 2. There were no significant effects of strain except that B6.A mice had more (*P* < 0.05) skin fat than B6 mice. Male mice had larger fat pads (*P* < 0.05) and greater fat content in the carcass and tail (*P* < 0.05) compared to females. Oil red staining revealed higher (*P* < 0.05) intramuscular fat content in the tibialis anterior muscle of male mice compared to female mice, but there were no effects of strain on this measure.

### 2.6. Plasma Lipids and Insulin

Data on the fasting levels of plasma lipids and insulin at the end of HFD study are presented in Table 3. The significant differences between the strains were observed only in males. Plasma FFA and LDL cholesterol were higher (*P* < 0.05) in B6.A males compared to B6 males. A similar trend was observed for TAG (*P*=0.054). Females tended to have lower levels of plasma lipids than males and the sex effects were significant (*P* < 0.05) for TAG, total cholesterol, and HDL cholesterol. Insulin levels were lower in females than males (*P* < 0.001), but there were no differences between the strains.

### 2.7. Glucose Tolerance

**Figure 5:**
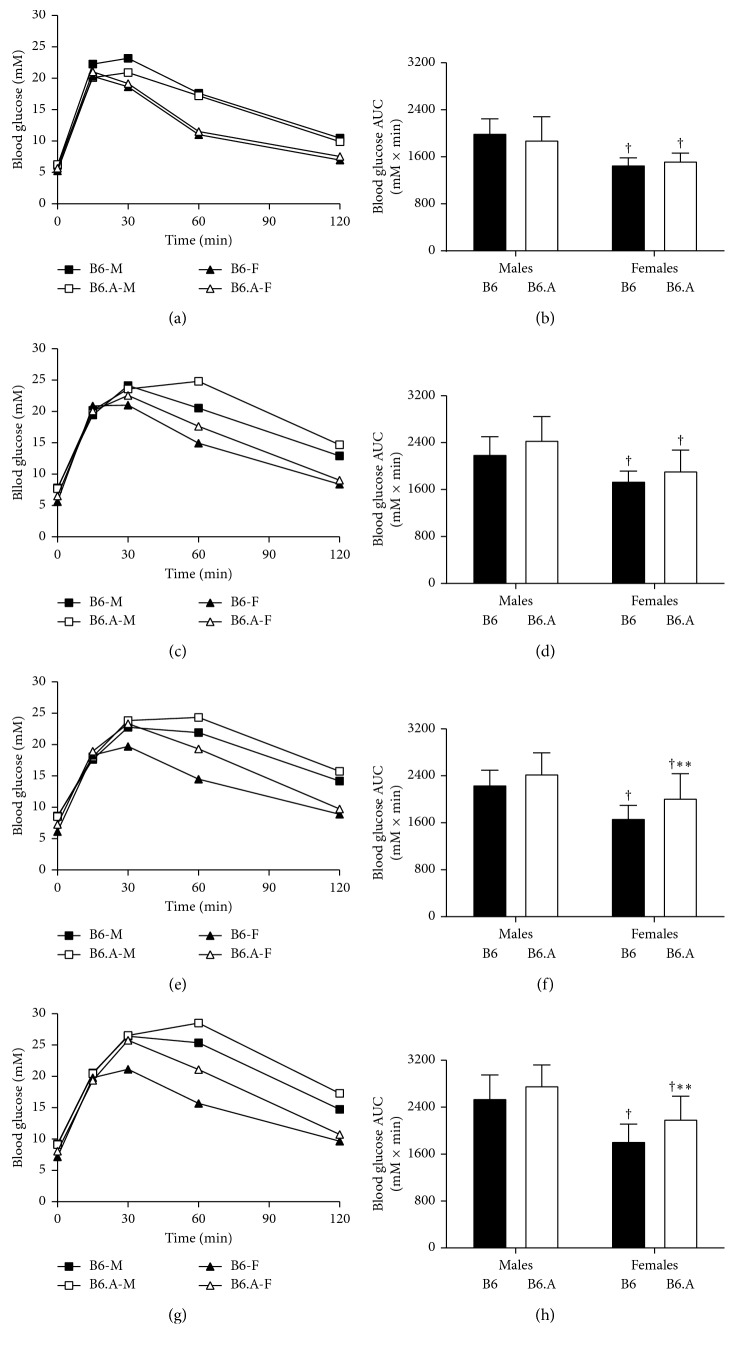
Glucose tolerance. Blood glucose concentrations (mM) and area under curve (AUC) during glucose tolerance tests consisting of the peritoneal bolus injection of glucose (2 g glucose/kg body weight) in C57BL/6J (B6) and B6.A-(rs3676616-D10Utsw1)/KjnB6 (B6.A) mice fed high carbohydrate diet (a, b) as well as after for 2 weeks (c, d), 6 weeks (e, f), and 12 weeks (g, h) of high fat diet feeding. Values are means (a c, e, and g) and means ± SD (b, d, f, and h). ^†^*P* < 0.001 males vs females; ^*∗∗*^*P* < 0.001, B6 vs B6.A strains.

Data on glucose tolerance in HFD study are shown in Figure 5. Fasting blood glucose levels increased (*P* < 0.01) during HFD feeding and were consistently higher (*P* < 0.001) in males than females independently of the strain. Blood glucose peaked from 15 min to 60 min after intraperitoneal injection of glucose and decreased afterwards. The blood glucose AUC was consistently greater in males compared to females (*P* < 0.001) and increased (*P* < 0.001) for both sexes during HFD feeding. B6 females had a lower (*P* < 0.05) blood glucose AUC than B6.A females after 6 and 12 weeks of HFD (Figures 5(f) and 5(h)). A similar trend was observed for males (*P*=0.07) at these time points. Blood glucose values 60 min after glucose injection did show a clear strain effect (*P* < 0.05) for both sexes as they were higher in B6.A mice compared to B6 mice.

### 2.8. Low CS Activity Is Associated with High Cellular Toxicity after Exposure to High Extracellular Palmitate Concentration

**Figure 6:**
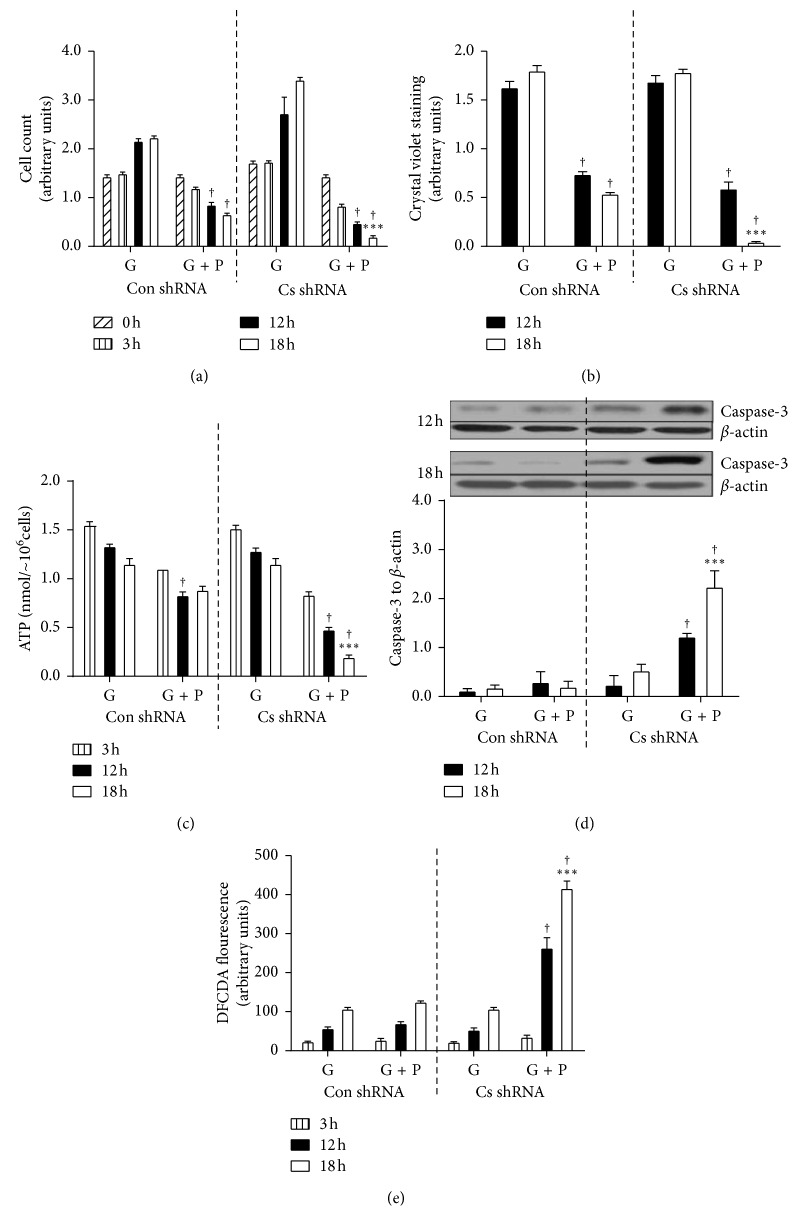
Palmitate-induced lipotoxicity in C2C12 muscle cells. C2C12 muscle cells were treated either with control shRNA (Con shRNA) or Cs shRNA which targeted *Cs* mRNA. Afterwards Con shRNA and Cs shRNA cells were incubated in the media containing 5.5 mM glucose (G) and/or 0.8 mM palmitate (P). (a) Cell proliferation was assessed using cell counting assay kit-8 (96992, Sigma, UK) and verified by (b) crystal violet staining; (c) cellular ATP concentrations were assessed using ATP calorimetric assay (ab83355, Abcam, Cambridge, UK); (d) levels of cleaved caspase-3 were assessed using immunoblotting; (e) production of reactive oxygen species was assessed by measuring 2′, 7′-dichlorodihydrofluorescein diacetate (H2DCFDA) fluorescence (values are means ± SEM). ^*∗∗*^*P* < 0.01, B6 vs B6.A strains. ^†^*p* < 0.001 G vs G + P; ^*∗∗∗*^*P* < 0.001, Con shRNA vs Cs shRNA.

Cell growth and stress markers were studied in C2C12 mouse muscle cells treated with Con shRNA and Cs shRNA which targeted *Cs* mRNA. Compared to Con shRNA cells, Cs shRNA cells showed lower Cs mRNA to *β*-actin mRNA ratio (0.022 ± 007 versus 0.081 ± 016, respectively, *P* < 0.001) and reduced CS activity (406.6 ± 115.5 versus 199.7 ± 72.4 *μ*mol/min/g protein, respectively, *P* < 0.001).  Figure 6 shows data on growth and metabolic stress of these cells cultured with and without palmitate.

**Figure 7:**
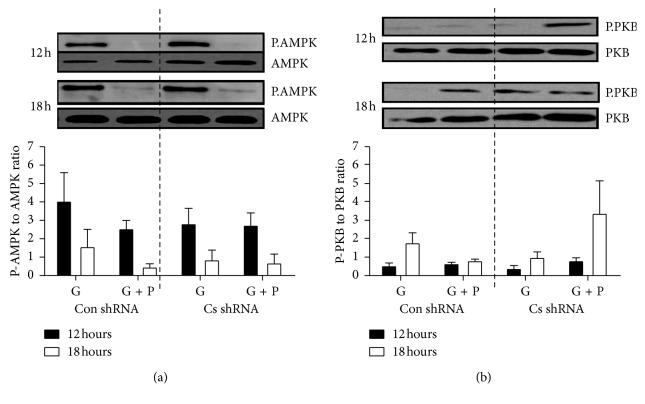
Palmitate-induced lipotoxicity in C2C12 muscle cells (Cont). C2C12 muscle cells were treated either with control shRNA (Con shRNA) or Cs shRNA which targeted Cs mRNA. Afterwards Con shRNA and Cs shRNA cells were incubated in the media containing 5.5 mM glucose (G) and/or 0.8 mM palmitate (P). (a) Effect of palmitate exposure on the phosphorylation of AMPK (Thr 172) in Con shRNA and Cs shRNA cells using western blot analysis. Quantification of immunoblots from six experiments. P.AMPK was normalized to AMPK. (b) Effect of palmitate exposure on the phosphorylation of PKB (Ser473) in Con shRNA and Cs shRNA cells. Quantification of immunoblots from six experiments. P.PKB was normalized to PKB. Control cells with 5.5 mM glucose were used as reference. Protein load: 25 *µ*g expression. Values are means ± SEM.

Under the standard conditions of high glucose total cell staining (indicating number of cells) tended to increase from 12 h to 18 h incubation though this increase was significant (*P* < 0.05) only for Con shRNA cells. Palmitate supplementation of the cell media caused a marked (*P* < 0.001) decrease in total cell staining, and this decrease was greater (*P* < 0.001) in Cs shRNA than Con shRNA cells after 18 h incubation. Caspase-3 to *β*-actin ratio increased (*P* < 0.001) only in Cs shRNA cells and only after palmitate supplementation. There were no significant differences between Con shRNA and Cs shRNA cells in levels of PKB, P-PKB, AMPK, or P-AMPK proteins. There were no significant changes in P-PKB to PKB ratio or the P-AMPK to AMPK ratio either Figure 7.

## 3. Discussion

The aim of the study was twofold. Firstly, we investigated if the A/J strain variant of H55N polymorphism impairs CS activity and metabolic health in mice fed HFD. Secondly, we tested if *Cs* knockdown promotes palmitate-induced apoptosis in muscle cells. In agreement with our first hypothesis, B6.A mice had low CS activity, tended to accumulate more subcutaneous fat, and were less tolerant to glucose compared to B6 mice when fed HFD. Some of the effects of the strain were sex-specific as B6.A males but not that females had higher levels of plasma TAG and LDL cholesterol compared to B6 mice. In agreement with our second hypothesis ∼50% knockdown of CS activity accelerated palmitate-induced apoptosis in C2C12 mouse muscle cells. In summary, the results of our study suggest that CS activity is of particular importance for metabolic health but affects neither energy balance nor preponderance to obesity in mice.

We studied B6 mice and congenic B6.A mice which carry the A/J allele in the telomeric locus of chromosome 10 on B6 strain background [17]. This locus includes the *Cs* gene, and we proposed that histidine replaced by asparagine at the residue 55 was the main cause of low CS activity in A/J strain [6]. Our current results support this hypothesis as B6.A mice had significantly lower CS activity than B6 mice in both the gastrocnemius muscle and heart. Earlier studies that examined the effects of the telomeric locus of chromosome 10 with a different set of congenic strains derived from the same pair of the founders showed that the presence of A/J allele at the locus confers obesity resistance [15, 16]. We did not reproduce this finding. We started HFD feeding when mice were 12-week old rather than 5-week old, used the diet with 45% not 60% energy from fat, and continued the feeding for 88 days instead of 100 days. However, it is unlikely that these methodological differences could explain the differences in the findings between the studies. The B6 strain showed similar weight gains in our study as in the two previous studies, but the congenic B6.A strain gained much more weight than the congenic mice in the studies of Burrage et al. [15] and Shao et al. [16]. It appears that the recombination between the B6 and A/J alleles in the B6.A strain occurred slightly closer to the telomere resulting in a shorter insert of the A/J allele, missing the gene that conferred obesity resistance of the congenic strain used by Shao et al. [16].

There were only minor differences in weight gain between two strains as B6 males tended to gain weight faster than B6.A males during the first two weeks of HFD. Nevertheless, B6.A females experienced more rapid worsening in glucose tolerance than B6 mice when fed HFD. A similar trend was observed in males. Fasting insulin levels did not differ between the strains suggesting that insulin resistance was not implicated in the differences in glucose tolerance between the strains or differences in insulin resistance were minor and/or less pronounced under the fasting conditions. Impaired fatty acid oxidation is often considered as the major cause of insulin resistance [1, 18]. Muscle cell cultures of diabetic patients showed low CS activity and reduced rate of complete palmitate oxidation compared to the cell cultures derived from the healthy volunteers [8, 9]. It has been hypothesized that an inborn defect in the CS enzyme can limit complete fatty acid oxidation in muscle cells and thus cause insulin resistance in type 2 diabetes [9]. However, diabetic patients present a heterogeneous population and multiple factors could contribute to low CS activity and impaired lipid metabolism. Interestingly, we have also observed elevated blood lipids in B6.A males and high skin fat content for both sexes of B6.A mice compared to B6 mice. These results support the contention about importance of CS activity for optimal lipid metabolism. We tested this hypothesis further using C2C12 muscle cells which show apoptosis when cultured in the media supplemented with high palmitate concentration [10]. This palmitate-induced apoptosis is sensitive to the rate of fatty oxidation as overexpression of CPT1 which promotes fatty acid oxidation and also reduces apoptosis in these muscle cells [10]. In agreement with our hypothesis the cells with CS knockdown showed greater apoptosis as reflected in lower viability and higher levels of cleaved caspase-3 after 18 h exposure palmitate compared to the control cells. It has been reported that treatment with shRNA targeting *Cs* also increased caspase-3 levels in mouse's inner ear cell line [19]. Interestingly, C2C12 muscle cells with reduced CS activity did not differ in viability from the control cells when cultured in high glucose media without palmitate supplementation. It appears that 50% CS knockdown induces only subtle changes in muscle cell function. PKB and AMPK phosphorylation were not affected suggesting that neither insulin signalling nor ATP levels were reduced. This can probably explain why B6 and B6.A mice do not differ in glucose tolerance at the baseline when they were fed high carbohydrate diet. Our previous study showed that C2C12 muscle cells with low CS activity accumulated more ceramide, but only when incubated in the media supplemented with high concentrations of palmitate [20]. Interestingly, hair cells, spiral ganglion neurons, and stria vascularis in cochleae of A/J mice show degeneration which is linked to *Cs* even when these mice are fed a high carbohydrate diet [17, 19]. It appears that cellular response to CS knockdown depends on the cell type [21]. Interestingly, CS activity decreases faster than the activity of other mitochondrial enzymes in lymphocytes of ageing humans, and these changes are even more apparent in obese individuals [22]. The consequences of these alterations in CS activity are unclear and need to be investigated further in view of the current findings suggesting that physiological mechanisms underlying glucose tolerance and obesity can be dissociated.

Neither MEI nor DEE differed between B6 and B6.A mice. Thermoregulation contributed significantly to DEE in our study as mice were housed in separate cages at an ambient temperature of ∼21°C rather than 30°C which is within the thermoneutral zone for mice [23, 24]. The A/J mice have somewhat impaired thermoregulation as they are not able to maintain body temperature at the ambient temperature of 15°C [13]. However, all mice maintained their body temperature and B6.A males tended to show even higher body temperature than B6 mice in our study. B6.A males also showed a slightly higher RMR compared to B6 males after 12 weeks of HFD feeding. This could be due to marginally greater fat free mass of B6.A males. Physical activity did not depend on the strain. It appears that low CS activity leads to neither deficient thermoregulation nor alterations in energy balance when mice are maintained at temperatures slightly below the thermoneutral zone.

We studied mice of both sexes. Females tended to gain weight at a slower rate and showed better glucose tolerance than males throughout the whole duration of HFD study. This could be at least partially due to higher levels of physical activity in females compared to males. Indeed, increased levels of physical activity can reverse impairments in muscle metabolism even in rats selected for low running capacity [25]. However, in contrast to males, female mice also show an increase in population of anti-inflammatory T cells in adipose tissue and no clear signs of low grade inflammation which is a key contributor to insulin resistance during HFD feeding [26, 27]. There is also evidence that brown adipose tissue (BAT) of female rats shows higher expression of numerous proteins involved in thermogenesis and fat oxidation as well as lower expression of proteins contributing to fat synthesis compared to male rats [28]. Thus, several factors might have contributed to better glucose tolerance in female mice compared to male mice. Interestingly, CS and HAD activity of both mitochondrial enzymes (CS and HAD) tended to be somewhat lower in the gastrocnemius muscle of females compared to males. It appears that small reduction in the mitochondrial enzyme activity does not have a significant impact on insulin resistance during HFD feeding which promotes insulin resistance in mice [29, 30].

In summary, low CS activity is associated with impaired glucose tolerance and abnormalities in lipid metabolism which are more prevalent in males compared to females, but affects neither weight nor fat gain in mice fed HFD.

## 4. Materials and Methods

### 4.1. Animals and Measurements

Firstly, we tested the hypothesis that low CS activity impairs metabolic health of B6.A and B6 mice fed HFD. All procedures concerning animal care and treatment were approved by the Ethical committee for the use of experimental animals of the University of Aberdeen and licensed by the UK Home Office (PPL 60/4366). For this study, males and females of C57BL/6J (B6) and congenic B6.A-(rs3676616-D10Utsw1)/KjnB6 (B6.A) strains were purchased from Harlan (Charles River Laboratories, Wilmington, Massachusetts, USA) and the Jackson Laboratories (Bar Harbour, ME, USA), respectively. Mice were maintained in a temperature controlled room (21 ± 1°C) under a 12-h to 12-h light to dark cycle. Mice were individually housed in standard cages with *ad libitum* water and standard chow (CRM pellets, SDS diets, U.K.). Sixteen male and 16 female mice of both B6 and B6.A strains were studied (total *n*=64). All mice were implanted intraperitoneally with body temperature transmitters (PDT-4000 E-Mitter; Mini Mitter Bend, OR, USA) under general anaesthesia (mixture of isoflurane and oxygen) at approximately 8 weeks of age. Mice were allowed at least 7 days to recover from the surgery before they were put on a low fat high carbohydrate diet (HCD, D12450B, 10% kcal fat, 70% kcal carbohydrate, Research Diets, New Brunswick, NJ, USA). Baseline measurements then started at the age of 10 weeks and were taken over a period of 2 weeks. At 12 weeks of age all mice were switched to the HFD (D12451, 45% kcal from fat, Research Diets, New Brunswick, NJ, USA). Body weight (BW) and food intake (FI) were monitored, just prior to lights off, 3 times a week (Mondays, Wednesdays, and Fridays) throughout the experimental protocol. During the baseline period and after 2, 6, and 12 weeks of HFD, fat mass, resting metabolic rate (RMR), and glucose tolerance (GT) were measured as described below. We also assessed daily energy expenditure (DEE) using the doubly labelled water technique at the baseline and after 12 weeks of HFD feeding.

Body temperature (Tb) and physical activity (PA) were recorded for a period of 7 days every 4–6 weeks throughout the experimental protocol by placing mice in their home cage onto transponder energizers (ER-4000 Receiver, Mini Mitter Company Inc., USA). The VitalViewTM Data Acquisition System (Mini Mitter Company, Inc, USA) was used to collect the data in 1 min intervals, and PA counts and Tb (°C) were calculated as daily means over the 7-day period.

After 13 weeks of HFD, mice were fasted for 4 h and euthanized by CO_2_ and blood samples were taken by cardiac puncture. For 8 out 16 randomly selected mice from each experimental group, heart and gastrocnemius muscle and tibialis anterior from the left hindlimb were excised and snap frozen in isopentane precooled by liquid nitrogen and stored at −80°C for analysis of mitochondrial enzymes or intramuscular fat. For the remaining 8 mice, fat pads, carcass, tail, skin (including subcutaneous fat), liver as well as thyroid, heart, lungs, gonads, kidneys, spleen, stomach, large intestine, small intestine, and brain were taken to perform chemical analysis of fat content.

### 4.2. Food Intake

Gross energy intake with food was assessed using similar methods as previously described [31]. Food intake was calculated from the difference between the food given and the food residue. Food samples were taken to determine the water content. The energy density of dry food and faeces was determined by Parr1281 oxygen bomb calorimetry (Parr Instrument, Moline, IL, USA). Metabolisable energy intake (MEI, kJ day^−1^) was calculated as follows:

(1) $$\begin{array}{c} \text{gross energy intake}=\text{dry food intake }\times \text{ food energy density},\\ \text{MEI}=\text{gross energy intake}-\text{dry faeces mass }\times \text{ faeces energy density}-\text{urinary energy loss}. \end{array}$$

Energy loss with urine was assumed to be 3% of gross energy intake.

### 4.3. Daily Energy Expenditure

We measured daily energy expenditure (DEE) using the doubly labelled water technique [32]. Animals were weighed to a precision of 0.01 g and injected intraperitoneally with approximately 0.20 g of water containing enriched ^18^O (27.8·atom%) and ^2^H (15.9·atom%). Syringes were weighed before and after administration (±0.0001 g) to calculate the mass of doubly labelled water injected. Blood samples were taken after 1 h of isotope equilibration to estimate initial isotope enrichments and were also collected from unlabelled animals to estimate the background isotope enrichments. Blood samples were immediately heat sealed into two 50 *μ*l glass capillaries and stored at room temperature. A final blood sample was taken approximately 48 h later to estimate isotope elimination rates. Capillaries that contained the blood samples were then vacuum distilled, and water from the resulting distillate was used to produce CO_2_ and H_2_. The isotope ratios ^18^O to ^16^O and ^2^H to ^1^H were analysed using gas source isotope ratio mass spectrometry (ISOCHROM*μ*GAS system and IsoPrime IRMS, Micromass, Manchester, UK). Three high-enrichment standards were analysed each day alongside the samples. Isotope enrichments were converted to CO_2_ production as previously explained [32].

### 4.4. Resting Metabolic Rate

Resting metabolic rate (RMR) of all measurements took place during the light phase between 0700 and 1600 as described previously [23]. Briefly, air was pumped (Charles Austin Pumps) through a sealed Perspex chamber within an incubator (INL-401N-010, Gallenkamp) set at 30°C, i.e., within the thermo-neutral zone for the mice [24]. Mass-flow controllers (MKS Instruments UK, Cheshire, UK) produced 500–700 ml O_2_/min. Air that leaves the animal chamber was dried by silica gel and 150 ml min^−1^ was passed through a gas analyser (Servomex 1100A or Servomex Xentra, Servomex Ltd, Crowborough, UK). Gas concentrations were measured continuously, and mean values were stored every 30 s for 3 hours. RMR final quantification was measured as the oxygen consumption over the lowest 20 consecutive values (10 min interval) and corrected for ambient temperature and pressure. Data then were converted to energy equivalents using an oxycalorific value of 21.117 J ml^−1^ O_2_, derived from the Weir equation [33].

### 4.5. Body Composition

Fat mass (FM) of mice was measured using dual energy X-ray absorptiometry (DEXA; PIXImus2 Series Densitometers with software version 1.46.007; GE Medical Systems Ultrasound and BMD, Bedford, UK). Mice were anesthetized by inhalation of a mixture of isoflurane and oxygen for the duration of the scan (∼3 min). The head was excluded from the analysis of body fat [34].

### 4.6. Tissue Fat

Extraction of tissue fat was carried out using the Soxhlet method. Dry organs, fat pads, and carcasses with tail were placed into extraction thimbles (FB59483; Fisher brand, London, UK) weighed and placed into the extraction tube of the Soxhlet apparatus. Diethyl ether (BH code 281326G, VWR, Poole, Dorset, UK) was dripped throughout the extraction thimble until the solvent was clear in the tube. Tissue fat mass was calculated by subtraction of dry mass of sample and thimble after and before extraction.

### 4.7. Intramuscular Fat

The transverse sections from the belly of the tibialis anterior muscle were cut at a thickness of 10 *μ*m with a cryotome (Leica CM1850UV) at −20°C. The sections were fixed in 4% formaldehyde (Sigma, UK) for 1 h, washed by deionized water before incubating the slides with a 6 mM Oil Red dye diluted in isopropanol for 30 minutes. Intensity of the sample staining was then assessed using Image J software (National Institute of Health, USA) by measuring the difference in intensities between red and blue colours. The staining of the background was subtracted from the sample staining.

### 4.8. Mitochondrial Enzymes

The enzyme activity was analysed as in our previous studies [6, 20]. Samples of gastrocnemius muscle and hearts were homogenized in 10 volumes of ice cold lysis buffer (50 mM Tris–HCl, 100 mM KHPO_4_, 2 mM ethylenediaminetetraacetic acid, 0.2% wt./vol bovine serum albumin (BSA), pH was adjusted to 7.0). The homogenates were shaken for 60 min and centrifuged at 13 000 g for 10 min. The supernatants were taken and the protein concentration was measured using the Bradford assay. Then, measurements of citrate synthase (CS) and *β*-hydroxyacyl-coenzyme (CoA) dehydrogenase (HAD) activity were carried out at room temperature of 21°C using the spectrophotometer (GENESYS 10 Bio UV-Vis, Thermo Fisher Scientific Inc., Waltham, MA, USA). For the CS assay, the molar extinction coefficient used was 13 600 M^−1^ cm^−1^ for CoA-5,5′-thiobis(2-nitrobenzoic acid) at 412 nm. The reaction reagent consisted of 100 mM triethanolamine-HCl, dithiobis(2-nitrobenzoic acid), 0.5 mM Triton-X (0.25% vol/vol), oxaloacetate, and 0.31 mM acetyl CoA with pH adjusted to 8.0. CS from a porcine heart was used as a standard (C3260-200UN, Sigma-Aldrich Company Ltd, Gillingham, Dorset, UK) for assay calibration. For HAD assay, the molar extinction coefficient used was 63,000 M^−1^ cm^−1^ for NADH at 340 nm. The reaction reagent consisted of 100 mM tetrasodium pyrophosphate, 0.23 mM NADH, and 0.24 mM acetoacetyl CoA with pH adjusted to 7.3. The 1000 *μ*L of reaction reagent included 20 *μ*L of muscle homogenate.

### 4.9. Glucose Tolerance

The overnight fasted mice were subject to an intraperitoneal glucose injection (2 g glucose (kg body wt)^−1^). Blood glucose was measured with a glucometer (ACCU-Chek Aviva, Roche, West Sussex, UK) using the whole blood samples taken from cut tail tips immediately before and at 15, 30, 60, and 120 min after the injection. The area under curve for changes in blood glucose over time or blood glucose AUC was calculated using Prism 5.0 software.

### 4.10. Plasma Lipids and Insulin

After heart puncture blood was collected into 2 ml containers and centrifuged (5702/R, Eppendorf, Hauppauge, USA) at 1500 g at 4°C for 10 min, plasma concentrations of free fatty acids (FFA), triacylglycerol (TAG), total cholesterol (TC, mM), and HDL cholesterol (HDL-C) were assessed using commercially available kits (Randox, Crumlin, U.K.), using the spectrophotometer (GENESYS 10 Bio UV-Vis, Thermo Fisher Scientific Inc., Waltham, MA, USA). LDL cholesterol (LDL-C) concentration was determined as previously described [35]. Plasma insulin concentrations were measured in duplicates using the enzyme-linked immunosorbent assay (insulin ELISA, # nr 10-1247-01, Mercodia, Sweden) and the spectrophotometric plate reader (Synergy HT Multi-Mode Microplate Reader, BioTek, UK).

### 4.11. Genotyping

The proximal boundary of the congenic region in the B6.A strain was genotyped in order to refine the recombination site. Genomic DNA was extracted from muscle tissue obtained of B6.A as well as B6 and A/J, to provide positive control, using the hot sodium hydroxide and Tris (HotSHOT) method [36] Genomic segments flanking four SNPs partitioning the region of interest and polymorphic between the B6 and A/J strains were amplified by PCR using pairs of primers provided in parenthesis; rs29356783 (forward-AAGAGGAAGAGCCGAAAAGG; reverse-TAGGCATGATCAAGCACGAG), rs232632450 (GGGAATCAAACCCAGATCCT; ATGTGGGTCCCAGAAATCAA), rs48666233 (TAAATTTCAGGCGAGCTGGT; GCCTTTTCTTTCCTCCGTCT) and rs45825880 (TCCTTGTCGAGCTCCTCCTA; CCTCTTGGGAGGAAACAAGG). The amplicons were afterwards digested using AluI (Thermo Scientific, UK), BtgI (New England Biolabs, UK), HpyCH4V (New England Biolabs, UK) or NciI (Fermentas Life Sciences, UK) restriction enzymes, respectively, following manufacturer recommendation. The digests were separated on 2% agarose gel, imaged, and inspected for presence of the B6 or A/J allele.

### 4.12. Cell Cultures and Experiments

C2C12 mouse muscle cells were used to investigate the effects of reduced CS activity on palmitate-induced lipotoxicity. The cells were cultured in growth medium containing 88% (vol/vol) Dulbecco's Modified Eagle's medium (DMEM), 5.5 mM glucose, 10% (vol/vol) fetal calf serum (FCS), and 2 mM glutamine in T75 cm^2^ flasks at 37°C and 5% CO_2_ as previously described [37]. Then, lentivirus-delivered stable gene silencing was applied to knock down *Cs* expression in the cells [38]. Pseudoviruses were produced by cotransfecting HEK293 cells with a plasmid carrying shRNA and the Mission lentiviral packaging mix (SH001, Sigma–Aldrich) containing plasmids expressing viral packaging genes and a heterologous viral envelope gene. We used shRNA (GCACCCAACATTTGAGTTATTCTCGAGAATAACTCAAATGTTGGGTGC) which targets the 3′untranslated region (UTR) of Cs mRNA (Cs shRNA) and control shRNA (Con shRNA) containing random sequence. The shRNA was delivered within the pLKO.1-puro vector containing the puromycin resistance marker. Virus was harvested in the culture supernatant at 72 h posttransfection, and transductions of C2C12 cells were carried out in the presence of 10 mg l^−1^ of polybrene. After transduction, cells were selected in 3 mg l^−1^ of puromycin for 3 days and then used in the experiments with palmitate incubation. For these experiments, 2 × 10^6^ cells were seeded in six well plates coated with extracellular matrix gel (E6909, Sigma-Aldrich, MO, USA) and containing 2 ml of the growth medium. When cells became confluent, the medium was changed to the differentiation medium containing 96% DMEM, 5.5 mM glucose, 2% horse serum, and 2 mM glutamine. The extent of shRNA-mediated Cs knockdown was assessed using the real time RT-PCR and measurements of CS activity using the spectrophotometric CS assay on Cs shRNA and Con shRNA cells. In experiments with glucose (G experiments), the differentiated Cs shRNA and Con shRNA myotubes were incubated in the differentiation media supplemented with 2% (wt/vol) fatty acid-free BSA and 1.2 mM L carnitine for up to 18 h. In experiments with mixture of glucose and palmitate (G + P experiments), 0.8 mM palmitate was added to the media. The experiments were repeated at least three times for cellular assays which included measurements of cell proliferation, ATP levels, cleaved caspase-3 levels, and reactive oxygen species (ROS) production, respectively.

### 4.13. Cell Proliferation

Cell proliferation was assessed using two independent methods. Firstly, cell counting assay kit-8 (96992, Sigma, UK) was used. ∼10^6^ of cells was lysed in 6 well plates, and light absorbance of lysates was measured at 450 nm using the spectrophotometer. Secondly, crystal violet staining was applied [39]. For these measurements, the cells were fixed by incubating them in 45% (vol/vol) formalin solution (#HT501128, Sigma-Aldrich, St-Louis, MI, USA) at room temperature for 30 min. The cells were then washed in phosphate buffered saline (PBS) and left to dry. The crystal violet solution was made by solubilizing 1 g of the dye powder (#C3886, Sigma-Aldrich, St-Louis, MI, USA) in 200 ml of 10% (vol/vol) acetic acid. 50 *μ*l of the dye solution was applied to the fixed cells. The cells were then washed three times in PBS to remove all the excess dye. Afterwards 100 *μ*l of the lysis buffer (50 mM tris hydroxymethyl aminomethane-hydrocloride, 1 mM ethylene diamine tetracetic acid, 1 mM ethylene glycol tetra acetic acid, 1% (vol/vol) Triton X-100, 0.1% (vol/vol) 2-mercaptoethanol, 10 mM *β*-glycerophosphate, 50 mM NaF, and 0.5 mM Na_3_VO_4_, 2% (vol/vol) protease inhibitor cocktail, pH of 7.5) was applied to lyse the cells. The light absorbance was measured in 20 *μ*l of the lysate in triplicates at 590 nm wavelengths (Synergy HT Multi-Mode Microplate Reader, BioTek, UK).

### 4.14. Cell Metabolism and Stress

#### 4.14.1. Real-Time RT-PCR

Cs shRNA and Con shRNA cells were homogenized in 1 ml of ice cold TRIZOL Reagent (Invitrogen Ltd, Paisley, UK) and RNA extracted using chloroform and isopropanol as described previously [6]. 2 *μ*g of RNA was then used for cDNA synthesis in 20 *μ*l reaction volume containing 50 mM Tris-HCl (pH 8.3), 75 mM KCl, 3 mM MgCl_2_, 0.5 mM dNTP Mix (0.5 mM each dATP, dGTP, dCTP and dTTP), 5 mM DTT, 150 ng of random primers, and 200 units of SuperScript™ III Reverse Transcriptase. Real time PCR was performed using Roche Lightcycler 480 II (Roche Diagnostics, Sussex, UK) and Multiplex Taqman assays for *Cs* as a target gene and *β-*actin as a reference gene in each sample. Three *μ*L of cDNA was added to 10 *μ*L of LightCycler 480 Probe Master (Roche), 0.2 *μ*L of TaqMan probe (Probe no. 100, Universal Probe Library), 0.2 *μ*L of forward and reverse primers (20 *μ*M) each, and 1 *μ*L of mouse *β*-actin probe dye VIC-MGB (Applied Biosystems, 4326317E). The mouse *Cs* intron spanning primers were designed using Universal Probe library software and purchased from Sigma-Genosys (forward primer: 5′-GGAAGGCTAAGAACCCTTGG-3′ and reverse primer: 5′-TCATCTCCGTCATGCCATAGT-3′) and the corresponding UPL probe (UPL probe #100) was used. The results were analysed using LightCycler® 480 software 1.5 and *Cs* was normalized to *β*-actin and presented as a ratio (ratio = (1 + *E*_Cs_)^−Ct(*Cs*)^/(1 + *E*_*β-*actin_)^−Ct(*β-*actin)^).

#### 4.14.2. Western Blotting

The cell media was removed and 100 *μ*l of the ice cold lysis buffer was applied. The lysed cells were transferred into the plastic tubes and snap frozen in the liquid nitrogen. Afterwards, the cells were thawed, homogenized using homogenizer (ULTRA-TURRAX, Rose Scientific, Edmonton, Canada), shaken for 60 min, and centrifuged at 13,000 g for 10 min at 4°C. The supernatants were taken, the protein concentration was measured using the Bradford assay, and samples with equal protein concentration were made in Laemmli buffer as in our previous studies [37]. 25 *μ*g of protein was usually loaded per lane on 10% polyacrylamide gel, separated using SDS-PAGE electrophoresis and transferred to polyvinylidene fluoride (PVDF) membrane. The membranes were washed with Tris buffered saline (TBS) containing 0.1% (vol/vol) Tween-20 (TBS-T buffer) before two hour incubation in the blocking buffer (5% (wt./vol) nonfat milk in TBS-T buffer). The membranes were incubated for 18 h at 4°C with a primary antibody at 1 : 1000 dilution (vol/vol) TBS-T buffer supplemented with 5% bovine serum albumin. All antibodies were from Cell Signalling Technology (Danvers, MA, USA). The primary antibodies of caspase-3 (#9662), phospho-Akt (Ser473) (#9271), Akt (#9272), AMPK (#2532), phospho-AMPK*α* (Thr172) (#2531) and *β*-actin (#4967s) were prepared at 1 : 1000 in blocking buffer (20 mM Tris buffer saline, 5% (wt./vol) bovine serum albumin, 0.1% (vol/vol) Tween). After incubation with a primary antibody, membranes were washed in TBS-T buffer and exposed for 2 h to HRP-conjugated secondary antibody (#7071) at 1 : 2000 dilution in the blocking buffer. The imaging of blots was performed using ECL reagent (Amersham Biosciences, Buckinghamshire, UK) and Fluor-SMax Imager (Bio-rad, Hertfordshire, UK). The images were quantified using Image J (NIH, USA) software.

### 4.15. Data Analysis

Data analysis was performed using SPSS and Prism 5.0 software. Values were expressed as means ± SD unless otherwise indicated. General linear models were used in the analysis. Analysis of covariance (ANCOVA), with body mass as a covariate, was applied to RMR, DEE, and MEI data. Repeated measures analysis of variance (ANOVA) was used in case of body weight (BW), body fat (BF), lean body mass (LBM), food intake (FI), mitochondrial enzyme activity, physical activity (PA), body temperature, and data on glucose tolerance. ANOVA was also used to assess effects of CS knockdown as well as type and duration of treatment on crystal violet staining and intracellular signalling in C2C12 cells. The *t*-test with a Bonferroni correction for multiple comparisons was used as a post hoc test. All the tests were two-tailed with the significance level set at *P* < 0.05.

## Figures and Tables

**Table 1:** Mitochondrial enzymes in tissues of C57BL/6J (B6) and B6.A-(rs3676616-D10Utsw1)/KjnB6 (B6.A) mice.

		Males		Females	
		B6	B6.A	B6	B6.A
CS	H	1834.6 ± 402.3	1063.6 ± 281.5^*∗∗*^	1736.4 ± 338.7	963.4 ± 205.9^*∗∗*^
	M^#^	750.9 ± 129.9	515.7 ± 96.0^*∗∗*^	644.7 ± 115.7	417.1 ± 113.0^*∗∗*^

HAD	H	151.7 ± 43.6	131.1 ± 28.6	137.7 ± 36.4	140.3 ± 21.7
	M^#^	12.7 ± 4.0	10.1 ± 2.6	8.4 ± 1.7	9.0 ± 3.7

Citrate synthase (CS) and *β* hydroxyacyl CoA dehydrogenase (HAD) activity (*μ*mol/min/g protein) in the gastrocnemius muscle (M) and heart (H) of mice was assessed using spectrophotometric assays for tissue homogenates after 12 weeks of high fat diet feeding. The values are means ± SD (*n*=8 each). B6 vs B6.A strains; ^*∗∗*^*P* < 0.001; ^#^males vs. females *P* < 0.05.

**Table 2:** Fat content in tissues of C57BL/6J (B6) and B6.A-(rs3676616-D10Utsw1)/KjnB6 (B6.A) mice after 12 weeks of high fat diet (HFD) feeding.

	Males		Females	
	B6	B6.A	B6	B6.A
Fat pads (g)^††^	5.818 ± 1.839	5.526 ± 1.231	4.397 ± 1.436	3.932 ± 1.290
Skin and subcutaneous fat (g)^*∗*^	2.614 ± 1.178	3.342 ± 1.448	2.073 ± 0.670	2.933 ± 0.726
Carcass and tail fat (g)^††^	2.559 ± 0.668	2.470 ± 0.563	1.893 ± 0.651	1.832 ± 0.493
Organ fat (g)	0.311 ± 0.084	0.397 ± 0.341	0.267 ± 0.157	0.222 ± 0.062
Liver fat (g)	0.182 ± 0.174	0.115 ± 0.081	0.081 ± 0.030	0.082 ± 0.030
TA muscle fat (au)^††^	15.64 ± 7.25	15.90 ± 5.59	11.39 ± 6.10	11.13 ± 3.64

Fat content of tissues was assessed using Soxhlet method for fat extractions. Intramuscular fat of the tibialis anterior (TA) muscle was evaluated using staining of the muscle cross sections with Oil Red dye with the subsequent quantification of the staining intensity. The values are means ± SD (*n*=8 each). B6 vs B6.A strains; ^††^*P* < 0.01, effect of sex; ^*∗*^*P* < 0.05, effect of strain.

**Table 3:** Fasting levels of plasma lipids and insulin in C57BL/6J (B6) and B6.A-(rs3676616-D10Utsw1)/KjnB6 (B6.A) mice after 12 weeks of high fat diet (HFD) feeding.

	Males		Females	
	B6	B6.A	B6	B6.A
Free fatty acids (mM) (*n*=8)	1.30 ± 0.38	1.69 ± 0.33^*∗*^	1.47 ± 0.27	1.48 ± 0.28
Triacylglycerol (mM)^†^ (*n*=16)	1.08 ± 0.29	1.37 ± 0.49	0.98 ± 0.29	1.04 ± 0.37
Total cholesterol (mM)^†^ (*n*=8)	3.96 ± 1.43	4.86 ± 0.77	3.93 ± 0.62	3.67 ± 0.82^†^
HDL cholesterol (mM)^*∗*^^†^ (*n*=8)	0.94 ± 0.14	0.79 ± 0.09	0.78 ± 0.08	0.72 ± 0.12
LDL cholesterol (mM) (*n*=8)	2.15 ± 1.18	3.54 ± 0.82^*∗*^	2.72 ± 0.57	2.29 ± 0.91^†^
Insulin (*μ*g L-1)^†††^ (*n*=7)	9.39 ± 3.27	7.19 ± 3.82	2.78 ± 0.54^††^	2.79 ± 0.30^†^

Plasma concentrations of free fatty acids (FFA), triacylglycerol (TAG), total cholesterol (TC, mM), and HDL cholesterol (HDL-C) were assessed using commercially available kits. LDL cholesterol (LDL-C) concentration was quantified as suggested by Friedewald et al. [35]. Plasma concentrations of insulin were measured using a standard enzyme-linked immunosorbent assay (insulin ELISA, # nr 10-1247-01, Mercodia, Sweden). The values are means ± SD. ^*∗*^*P* < 0.05, B6 vs B6.A strain; ^†^*P* < 0.05, ^††^*P* < 0.01, ^†††^*P* < 0.001 males vs females.

## Data Availability

The data used to support the findings of this study are available from the corresponding author upon request.

## References

[1] Houmard J. A. (2008). Intramuscular lipid oxidation and obesity. *American Journal of Physiology-Regulatory, Integrative and Comparative Physiology*.

[2] Powers S. K., Wiggs M. P., Duarte J. A., Zergeroglu A. M., Demirel H. A. (2012). Mitochondrial signaling contributes to disuse muscle atrophy. *American Journal of Physiology-Endocrinology and Metabolism*.

[3] Kristensen J. M., Skov V., Petersson S. J. (2014). A PGC-1*α*- and muscle fibre type-related decrease in markers of mitochondrial oxidative metabolism in skeletal muscle of humans with inherited insulin resistance. *Diabetologia*.

[4] Larsen S., Nielsen J., Hansen C. N. (2012). Biomarkers of mitochondrial content in skeletal muscle of healthy young human subjects. *Journal of Physiology*.

[5] Jacobs R. A., Díaz V., Meinild A.-K., Gassmann M., Lundby C. (2013). The C57Bl/6 mouse serves as a suitable model of human skeletal muscle mitochondrial function. *Experimental Physiology*.

[6] Ratkevicius A., Carroll A. M., Kilikevicius A. (2010). H55N polymorphism as a likely cause of variation in citrate synthase activity of mouse skeletal muscle. *Physiological Genomics*.

[7] Kilikevicius A., Venckunas T., Zelniene R. (2013). Divergent physiological characteristics and responses to endurance training among inbred mouse strains. *Scandinavian Journal of Medicine and Science in Sports*.

[8] Gaster M., Rustan A. C., Aas V., Beck-Nielsen H. (2004). Reduced lipid oxidation in skeletal muscle from type 2 diabetic subjects may be of genetic origin: evidence from cultured myotubes. *Diabetes*.

[9] Ørtenblad N., Mogensen M., Petersen I. (2005). Reduced insulin-mediated citrate synthase activity in cultured skeletal muscle cells from patients with type 2 diabetes: evidence for an intrinsic oxidative enzyme defect. *Biochimica et Biophysica Acta (BBA) - Molecular Basis of Disease*.

[10] Henique C., Mansouri A., Fumey G. (2010). Increased mitochondrial fatty acid oxidation is sufficient to protect skeletal muscle cells from palmitate-induced apoptosis. *Journal of Biological Chemistry*.

[11] Han D.-H., Hancock C., Jung S.-R., Holloszy J. O. (2009). Is “fat-induced” muscle insulin resistance rapidly reversible?. *American Journal of Physiology-Endocrinology and Metabolism*.

[12] Leibowitz S. F., Alexander J., Dourmashkin J. T., Hill J. O., Gayles E. C., Chang G.-Q. (2005). Phenotypic profile of SWR/J and A/J mice compared to control strains: possible mechanisms underlying resistance to obesity on a high-fat diet. *Brain Research*.

[13] Kus V., Prazak T., Brauner P. (2008). Induction of muscle thermogenesis by high-fat diet in mice: association with obesity-resistance. *American Journal of Physiology-Endocrinology and Metabolism*.

[14] Singer J. B., Hill A. E., Burrage L. C. (2004). Genetic dissection of complex traits with chromosome substitution strains of mice. *Science*.

[15] Burrage L. C., Baskin-Hill A. E., Sinasac D. S. (2010). Genetic resistance to diet-induced obesity in chromosome substitution strains of mice. *Mammalian Genome*.

[16] Shao H., Burrage L. C., Sinasac D. S. (2008). Genetic architecture of complex traits: large phenotypic effects and pervasive epistasis. *Proceedings of the National Academy of Sciences*.

[17] Johnson K. R., Gagnon L. H., Longo-Guess C., Kane K. L. (2012). Association of a citrate synthase missense mutation with age-related hearing loss in A/J mice. *Neurobiology of Aging*.

[18] Cooney G. J., Thompson A. L., Furler S. M., Ye J., Kraegen E. W. (2002). Muscle long-chain acyl CoA esters and insulin resistance. *Annals of the New York Academy of Sciences*.

[19] Han X., Ge R., Xie G. (2015). Caspase-mediated apoptosis in the cochleae contributes to the early onset of hearing loss in A/J mice. *ASN Neuro*.

[20] Gabriel B. M., Al-Tarrah M., Alhindi Y. (2017). H55N polymorphism is associated with low citrate synthase activity which regulates lipid metabolism in mouse muscle cells. *PLoS One*.

[21] Lin C. C., Cheng T. L., Tsai W. H. (2012). Loss of the respiratory enzyme citrate synthase directly links the Warburg effect to tumor malignancy. *Scientific Reports*.

[22] Capková M., Houstek J., Hansíková H., Hainer V., Kunesová M., Zeman J. (2002). Activities of cytochrome c oxidase and citrate synthase in lymphocytes of obese and normal-weight subjects. *International Journal of Obesity*.

[23] Speakman J. R. (2013). Measuring energy metabolism in the mouse-theoretical, practical, and analytical considerations. *Frontiers in Physiology*.

[24] Ravussin Y., LeDuc C. A., Watanabe K., Leibel R. L. (2012). Effects of ambient temperature on adaptive thermogenesis during maintenance of reduced body weight in mice. *American Journal of Physiology-Regulatory, Integrative and Comparative Physiology*.

[25] Lessard S. J., Rivas D. A., Stephenson E. J. (2011). Exercise training reverses impaired skeletal muscle metabolism induced by artificial selection for low aerobic capacity. *American Journal of Physiology-Regulatory, Integrative and Comparative Physiology*.

[26] Pettersson U. S., Waldén T. B., Carlsson P.-O., Jansson L., Phillipson M. (2012). Female mice are protected against high-fat diet induced metabolic syndrome and increase the regulatory T cell population in adipose tissue. *PLoS One*.

[27] Hotamisligil G. S. (2006). Inflammation and metabolic disorders. *Nature*.

[28] Choi D. K., Oh T. S., Choi J.-W. (2011). Gender difference in proteome of brown adipose tissues between male and female rats exposed to a high fat diet. *Cellular Physiology and Biochemistry*.

[29] Hancock C. R., Han D.-H., Chen M. (2008). High-fat diets cause insulin resistance despite an increase in muscle mitochondria. *Proceedings of the National Academy of Sciences*.

[30] Han D.-H., Hancock C. R., Jung S. R., Higashida K., Kim S. H., Holloszy J. O. (2011). Deficiency of the mitochondrial electron transport chain in muscle does not cause insulin resistance. *PLoS One*.

[31] Wu S.-H., Zhang L.-N., Speakman J. R., Wang D.-H. (2009). Limits to sustained energy intake. XI. A test of the heat dissipation limitation hypothesis in lactating Brandt’s voles (*Lasiopodomys brandtii*). *Journal of Experimental Biology*.

[32] Speakman J. R., Król E. (2005). Comparison of different approaches for the calculation of energy expenditure using doubly labeled water in a small mammal. *Physiological and Biochemical Zoology*.

[33] Weir J. B. d. V. (1949). New methods for calculating metabolic rate with special reference to protein metabolism. *Journal of Physiology*.

[34] Hambly C., Adams A., Fustin J.-M., Rance K. A., Bünger L., Speakman J. R. (2012). Mice with low metabolic rates are not susceptible to weight gain when fed a high-fat diet. *Obesity Research*.

[35] Friedewald W. T., Levy R. I., Fredrickson D. S. (1972). Estimation of the concentration of low-density lipoprotein cholesterol in plasma, without use of the preparative ultracentrifuge. *Clinical Chemistry*.

[36] Truett G. E., Heeger P., Mynatt R. L., Truett A. A., Walker J. A., Warman M. L. (2000). Preparation of PCR-quality mouse genomic DNA with hot sodium hydroxide and tris (HotSHOT). *Biotechniques*.

[37] Watt K. I., Jaspers R. T., Atherton P. (2010). SB431542 treatment promotes the hypertrophy of skeletal muscle fibers but decreases specific force. *Muscle and Nerve*.

[38] Martin-Granados C., Philp A., Oxenham S. K., Prescott A. R., Cohen P. T. W. (2008). Depletion of protein phosphatase 4 in human cells reveals essential roles in centrosome maturation, cell migration and the regulation of Rho GTPases. *The International Journal of Biochemistry and Cell Biology*.

[39] Gherzi R., Trabucchi M., Ponassi M., Gallouzi I.-E., Rosenfeld M. G., Briata P. (2009). Akt2-mediated phosphorylation of Pitx2 controls Ccnd1 mRNA decay during muscle cell differentiation. *Cell Death and Differentiation*.

