# Corrigendum to “Low Citrate Synthase Activity Is Associated with Glucose Intolerance and Lipotoxicity”

**DOI:** 10.1155/2019/9153809

**Published:** 2019-08-01

**Authors:** Yosra Alhindi, Lobke M. Vaanholt, Mustafa Al-Tarrah, Stuart Gray, John R. Speakman, Catherine Hambly, Bader S. Al-Anazi, Brendan M. Gabriel, Arimantas Lionikas, Aivaras Ratkevicius

**Affiliations:** ^1^Clinical Pharmacy Department, Pharmacy Collage, Umm Al-Qura University, Makkah, Saudi Arabia; ^2^Institute of Biological and Environmental Sciences, University of Aberdeen, Aberdeen, UK; ^3^School of Medicine, Medical Sciences and Nutrition, University of Aberdeen, Aberdeen, UK; ^4^Functional Genomics Unit, Dasman Diabetes Institute, Kuwait City, Kuwait; ^5^Institute of Cardiovascular and Medical Sciences, University of Glasgow, Glasgow, UK; ^6^Karolinska Institute, Department of Physiology and Pharmacology, Integrative Physiology, Stockholm, Sweden; ^7^Department of Applied Biology and Rehabilitation, Lithuanian Sports University, Sporto 6, Kaunas LT 44221, Lithuania

In the article titled “Low Citrate Synthase Activity Is Associated with Glucose Intolerance and Lipotoxicity” [1], there was a missing affiliation for the third author. The corrected authors' list and affiliations are shown above.
